# The endocytic trafficking pathway of oncogenic papillomaviruses

**DOI:** 10.1016/j.pvr.2019.03.004

**Published:** 2019-04-01

**Authors:** Snježana Mikuličić, Luise Florin

**Affiliations:** Institute for Virology and Research Center for Immunotherapy (FZI), University Medical Center of the Johannes Gutenberg-University Mainz, Augustusplatz, 55101, Mainz, Germany

**Keywords:** Virus, Entry receptor complex, Tetraspanin, Annexin, Endocytosis, Trafficking

## Abstract

Over the last two decades many host cell proteins have been described to be involved in the process of infectious entry of oncogenic human papillomaviruses (HPV). After initial binding and priming of the capsid, a sequence of events on the cell surface precedes the formation of the HPV entry platform. It has been shown that the virus-associated entry complex consists of membrane organizers, tetraspanins CD151 and CD63, and their associated partner proteins such as integrins, growth factor receptors, and the annexin A2 heterotetramer. Further recruitment of cytoplasmic factors such as the obscurin-like protein 1 and actin results in a non-canonical clathrin-independent endocytosis of the virus. Internalized viruses are then routed to multivesicular bodies for capsid disassembly. This early trafficking again involves annexins, and tetraspanin proteins. In this review, we summarize the current knowledge about HPV16 endocytosis and the subsequent endosomal trafficking. Moreover, we propose a model on how tetraspanins and annexins organize the spatial accumulation of HPV16-associated molecules, the recruitment of cytoplasmic trafficking factors, and the L2 membrane penetration to trigger virus entry.

## 1 The HPV16 entry receptor complex

**Fig. 1:**
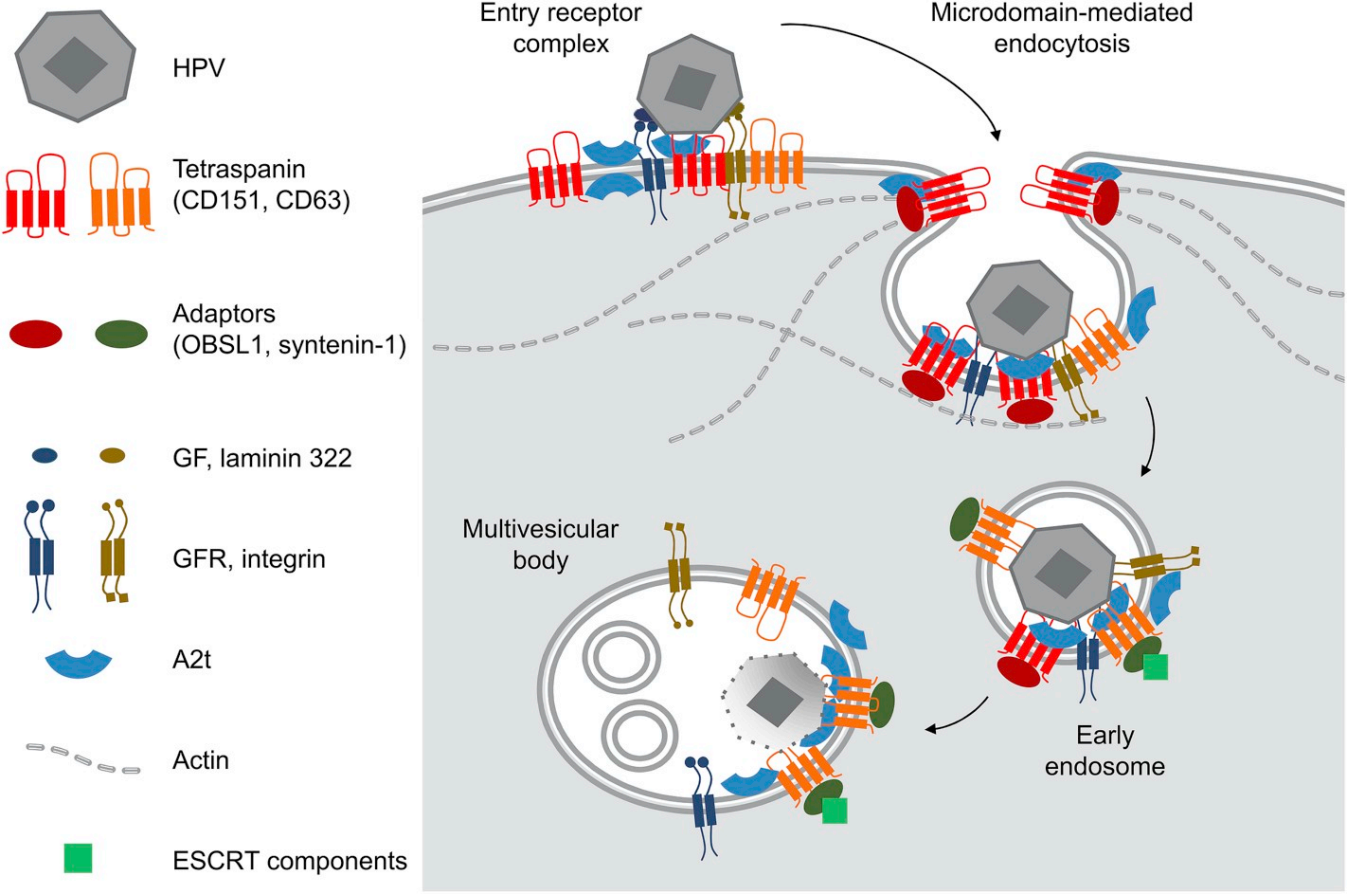
**Schematic diagram of HPV16 endocytosis and trafficking to multivesicular bodies.** After priming and activation of signaling cascades, the HPV16 capsid interacts with the entry receptor complex composed of growth factor receptors, laminin-binding integrins, tetraspanins and annexins. Upon HPV binding to this complex, a non-canonical and clathrin independent endocytic process is initiated, which involves actin, the cytoskeletal adaptor protein OBSL1, the annexin A2 heterotetramer and tetraspanin CD151. Internalized viruses are then routed to acidified multivesicular bodies. This early trafficking again involves annexins, and a complex of tetraspanin CD63, syntenin-1 and ESCRT components. Therefore, tetraspanins and annexins might organize the spatial accumulation of HPV16-associated molecules and recruitment of cytoplasmic trafficking factors until capsid disassembly and L2 membrane insertion/penetration ensures virus interaction with cytoplasmic partner proteins and subsequent trafficking towards and into the nucleus.

Oncogenic human papillomaviruses (HPV) of the genus alpha, such as HPV16, HPV18, and HPV31, enter keratinocytes via a pathway that depends on a coordinated sequence of events. It includes wounding of the mucosa, virus attachment to the extracellular matrix, followed by binding to primary and secondary receptor complexes, modifications of L1 and L2 capsid proteins and cell surface components as well as triggering signaling cascades (for review see M. Ozbun in the same issue). These extracellular events lead to the association of the viral capsid with the entry receptor complex (Fig. 1). A number of studies revealed the involvement of distinct cellular proteins as secondary HPV16-binding partners: laminin-binding integrin complexes [1,2], growth factor receptors (GFR) [3,4], the phospholipid-binding protein annexin A2 [4,5], and tetraspanins [2,6,7]. Co-immunoprecipitation studies demonstrated the physical interaction of HPV16 capsids with integrin α6 [1], epidermal and keratinocyte GFR [3], annexin A2 [4,5], and tetraspanins [8]. High diversity of HPV16 receptor candidates and the specific capability of tetraspanins to concentrate membrane proteins at particular sites proposes functional microdomains as the second receptor complexes [9]. Whether the secondary entry complex harbors additional HPV16 entry components remains to be determined.

## 2 HPV16 endocytosis

After the formation of the HPV-associated entry platforms, the capsids undergo internalization via endocytosis. For many years, the mode of HPV16 internalization has been contradictorily discussed. The implementation of siRNA-mediated depletion and overexpression of dominant-negative key players of canonical endocytic pathways such as clathrin, caveolin, and dynamin, unveiled the mechanism of HPV16 internalization into keratinocytes [6,10]. It became clear that HPV16 infects different cell types primarily via a less characterized endocytic mechanism [6,7]. This endocytic pathway of HPV16 depends on the reorganization of the actin cytoskeleton and shares characteristics to macropinocytosis [10]. Further investigations revealed that HPV16 endocytosis depends on the tetraspanin CD151 [2,6], the cytoskeletal adaptor obscurin-like 1 (OBSL1) [11], and the phospholipid-binding protein annexin A2 [4,5]. Subsequent studies showed that HPV16, 18, and 31 share similar requirements for entry suggesting that not only HPV16 but probably all HPV types of the alpha genus use this endocytosis pathway [7].

Tetraspanins are plasma membrane master organizers due to their ability to associate laterally with each other or various interaction partners. Tetraspanin CD151, a critical player in the HPV16 uptake, regulates the activities of associated laminin-binding integrins and directs HPV-binding molecules and functional virus entry factors [1,2]. Various CD151 mutants incompetent in binding integrins or other tetraspanins decreased HPV16 endocytosis, demonstrating that functional CD151-integrin and CD151-tetraspanin complexes are necessary during virus internalization. Furthermore, recovery experiments using C-terminally truncated CD151 emphasized the importance of the cytoplasmic domain in virus entry by its inability to restore HPV16 disassembly and infection in CD151-depleted cells [2]. This suggests CD151-integrin complex as a functional unit where CD151 uses its C-terminal tail to link the integrins to intracellular pathways [2]. Accordingly, a cytopermeable peptide containing the sequence of CD151 C-terminal tail is able to reduce HPV16 infection and capsid disassembly most likely by blocking interactions between CD151 and cytoplasmic interaction partners [12]. Together these studies stress the function of tetraspanins as organizers of the HPV entry platform as they direct co-factors into distinct microdomains at the plasma membrane to mediate endocytosis and trafficking processes of oncogenic HPV types.

Moreover, tetraspanin microdomains are connected to the actin cytoskeleton [11] which enables virus internalization. Actin acts as a separator of virus-filled endocytic pits and the plasma membrane [10]. In addition, actin was found to colocalize with the proviral cytoskeletal adaptor protein OBSL1 [11]. Epithelial cells deficient in OBSL1 exhibited diminished endocytosis and disassembly of virus particles as well as strongly reduced infection rates [11]. Immunofluorescence studies with OBSL1 confirmed its association with both capsid proteins and CD151 at the plasma membrane, while co-immunoprecipitations demonstrated the physical interaction of OBSL1 with minor capsid protein L2 suggesting an additional role of OBSL1 in L2-mediated steps of virus trafficking towards the nucleus. Alternatively, OBSL1 might act as a linker between L2, CD151 and the actin cytoskeleton, thereby enabling efficient HPV16 internalization.

The annexin A2 heterotetramer (A2t) is an additional relevant regulator of HPV16 infection. This complex localizes at both leaflets of the plasma membrane and is composed of two annexin A2 (AnxA2) monomers and a S100A10 dimer [4,5]. As tetraspanins, AnxA2 monomer and A2t complex, play important roles in membrane domain organization, cytoskeletal membrane dynamics, endocytosis, vesicular trafficking and exocytosis [13]. Moreover, the HPV16 capsid proteins L1 and L2 are able to physically interact with A2t subunits proposing A2t as a component of the HPV16 entry receptor complex [4,5]. The findings that cell treatment with AnxA2-targeting antibody and cellular depletion of A2t resulted in a significant reduction of HPV16 internalization and infection support this notion [4,5]. Besides, it was described that exposure to HPV16 induces EGFR-Src signaling cascade resulting in AnxA2 phosphorylation and consequently the translocation of the A2t complex to the outer leaflet of the plasma membrane [4], a process that may support complex formation of annexin, the HPV capsid, and EGFR. Moreover, the potency of A2t to connect actin filaments with the plasma membrane proteins allows actin-mediated membrane remodeling [13] which might lead to membrane curvature and vesicle scission at virus entry sites. Therefore, a scenario in which AnxA2 cooperates with tetraspanins in organizing the HPV entry platforms and link them to the actin cytoskeleton is plausible.

## 3 Early intracellular trafficking of HPV16

After virus uptake via tetraspanin and annexin-enriched microdomains, internalized virus particles are routed in association with tetraspanin CD63 and A2t to multivesicular bodies (MVBs) for capsid disassembly [8]. These early steps in HPV intracellular trafficking depend on various trafficking mediators including, syntenin-1 and components of the ESCRT machinery such as ALIX and VPS4 [8,14].

Different HPV types were found in colocalization with tetraspanin CD63, on the plasma membrane and in intracellular vesicles [6,8]. Co-immunoprecipitation studies detected the physical interaction of CD63 with HPV16 L1 [8]. CD63 depletion as well as cytoplasmic delivered peptides containing the C-terminal tails of CD63 resulted in a significant decrease of disassembled virus capsids [12]. More specifically, the early trafficking and transport of HPV16 from virus-containing early endosomes to MVBs is reliant on CD63-syntenin-1 complexes formed at HPV-containing endosomes [8]. Imaging and interaction analyses combined with expression recovery experiments using various CD63-/syntenin-1 mutants determined critical domains required for CD63/syntenin-1 complex formation and HPV capsid disassembly [8]. The analyses also identified the syntenin-1-interacting ESCRT protein ALIX as critical for HPV infection and CD63-syntenin-1-ALIX complex formation as a prerequisite for intracellular transport [8]. Additional ESCRT components might be part of this HPV transport complex [8,14]. These cumulative findings suggest CD63 as a critical linker between internalized viral particles and the trafficking machinery (Fig. 1).

Interestingly, HPV entry induces an increase in CD63/AnxA2 colocalization in endosomes [15] indicating cointernalization of the tetraspanin and A2t from the plasma membrane and/or fusion of internalized entry platforms with CD63 positive endosomal compartments. In this study, the authors stated that the depletion of A2t inhibited capsid uncoating while no effect on virus endocytosis was observed. Virus retention within early endosomes and disrupted virus trafficking to the MVBs in annexin A2 or S100A10knockout cells suggests the importance of annexin subunits in HPV trafficking [15]. Alternatively, A2t depletion might result in virus endocytosis into a non-infectious pathway that also leads to changes in intracellular transport, sorting and processing of the virus capsid. Moreover, the ability of A2t to bind to the minor capsid protein L2 [5] and to flip between the membrane leaflets implicates a scenario in which annexin assists in L2 membrane penetration during HPV entry, a prerequisite for successful delivery of the viral DNA into the nucleus and consequently infection.

## 4 Conclusions

Oncogenic HPV types of the genus alpha use a complex network of proteins for their endocytosis and intracellular transport that is organized by a specific subset of tetraspanins, annexins, and their partner proteins such as integrins and GFRs. Moreover, recruited adaptor proteins link the virus-associated membrane platform to the actin cytoskeleton during endocytosis and subsequently to the intracellular trafficking machinery. During these steps of HPV entry, the composition of tetraspanins and annexin A2 may change constantly to modulate diverse functions of the entry and trafficking platforms thereby triggering signaling events, membrane invagination, vesicle transport and vesicle maturation which leads to virus capsid disassembly (Fig. 1). Apart from a role in HPV trafficking, A2t might assist in L2 membrane penetration during HPV entry. Defining other, not yet unveiled components of the HPV16 entry platform will provide novel insights into the HPV16 propagation stages from the plasma membrane to the nucleus.

## Competing interests

The authors declare that the review was conducted in the absence of any commercial or financial relationships that could be construed as a potential conflict of interest.

## Abbreviations

A2t: annexin A2 heterotetramer
AnxA2: annexin A2
CD: cluster of differentiation
EGFR: epidermal growth factor receptor
ESCRT: endosomal sorting complexes required for transport
GFR: growth factor receptor
HPV: human papillomavirus
MVB: multivesicular bodies
OBSL1: obscurin-like protein 1
